# Short-term blood pressure variability assessed by ambulatory blood pressure monitoring as a predictor of subclinical target organ damage

**DOI:** 10.3389/fcvm.2026.1881263

**Published:** 2026-07-15

**Authors:** Yanling Hong, Sixin Xie, Huiqiong Jiang

**Affiliations:** Cardiac Function Examination Room, Quanzhou First Hospital (The First Affiliated Hospital of Fujian Medical University, Quanzhou), Quanzhou, Fujian Province, China

**Keywords:** ambulatory blood pressure monitoring, blood pressure variability, predictive value, retrospective study, subclinical target organ damage

## Abstract

**Background:**

Short-term blood pressure variability (BPV) from 24-h ambulatory blood pressure monitoring (ABPM) lacks evidence for predicting subclinical target organ damage (TOD).

**Objective:**

The study assessed BPV from ABPM for subclinical TOD prediction in hypertension.

**Methods:**

This retrospective study screened 180 essential hypertension patients at Quanzhou First Hospital between January 2023 and December 2025, and 176 patients were enrolled after exclusion. All participants underwent standardized 24-h ABPM examination. Several short-term BPV parameters were calculated, including standard deviation (SD), coefficient of variation (CV), and average real variability (ARV) for systolic blood pressure (SBP) and diastolic blood pressure (DBP) across 24-h, daytime, and nighttime periods. Patients were allocated into a TOD group and a non-TOD group based on the presence or absence of subclinical TOD. Multivariate logistic regression was used to assess BPV indices as independent predictors of subclinical TOD. Receiver operating characteristic (ROC) curve analysis assessed the predictive performance of these indices with the area under the curve (AUC) calculated.

**Results:**

Among 176 patients, 94 were assigned to the TOD group and 82 to the non-TOD group, with balanced baseline characteristics (all *P* > 0.05). The TOD group showed significantly higher 24-h, daytime, and nighttime BP SD, CV, and ARV (all *P* < 0.001). Multivariate logistic regression identified nighttime SBP SD, 24-h SBP ARV, and nighttime SBP ARV as independent predictors of subclinical TOD (all *P* < 0.05). ROC analysis showed nighttime SBP SD had an AUC of 0.837 (95% CI: 0.773–0.901), cutoff 12.25 mmHg, sensitivity 89.4%, specificity 78.0%, outperforming other indices (all *P* < 0.05). Subgroup analyses confirmed nighttime SBP SD independently predicted cardiac, vascular, and renal TOD, while 24-h SBP ARV predicted cardiac and vascular injury (all *P* < 0.05).

**Conclusion:**

Nighttime SBP variability (SD) from ABPM independently predicts subclinical TOD and guides organ protection in hypertension.

## 1 Introduction

Hypertension remains the leading risk factor driving the global burden of cardiovascular morbidity and mortality. Its pathological impact extends far beyond sustained elevations in blood pressure, and lies more prominently in the progressive injury inflicted on vital target organs including the heart, brain, and kidneys as a result of long-term abnormal blood pressure profiles (1, 2). Target organ damage (TOD) serves as a critical intermediate step linking hypertension to the full spectrum of the cardiovascular disease continuum. Early detection and timely intervention of subclinical TOD therefore carry substantial clinical importance in slowing disease progression and optimizing long-term patient outcomes (3). Conventional strategies for hypertension management have long centered on the control of clinic blood pressure or mean arterial pressure levels. However, a growing body of evidence in recent years has demonstrated (4) that blood pressure variability (BPV)—defined as the magnitude of blood pressure fluctuations over a defined period—also carries independent prognostic value regardless of baseline blood pressure levels, establishing itself as a valuable marker for predicting the occurrence of TOD and subsequent cardiovascular events.

Based on the time frame of measurement, BPV can be categorized into short-term, medium-term, and long-term variability. Short-term BPV generally describes fluctuations in blood pressure within a 24-h period, most commonly assessed using 24-h ambulatory blood pressure monitoring (ABPM). Several quantitative indices are routinely adopted for this purpose, such as standard deviation (SD), coefficient of variation (CV), and average real variability (ARV) (5, 6). Regarded as the gold standard for evaluating short-term BPV, ABPM enables comprehensive recording of blood pressure changes during a patient's daily activities, capturing distinct fluctuation patterns during daytime wakefulness and nighttime sleep. In doing so, it supplies far richer blood pressure–related information for clinical practice than conventional clinic blood pressure measurements (7). When compared with single clinic readings, ABPM not only offers a more accurate reflection of a patient's true blood pressure burden but also identifies circadian rhythm abnormalities and short-term fluctuations. Increasing attention has been directed toward the predictive performance of these ABPM-derived parameters in estimating the risk of TOD (8).

The pathophysiological mechanisms underlying short-term BPV involve multiple interconnected pathways. Dysfunction of the autonomic nervous system stands out as a key driver of unstable blood pressure. Elevated sympathetic nerve activity, together with reduced vagal tone, disrupts normal cardiovascular homeostatic regulation, thereby amplifying blood pressure fluctuations (9). In addition, increased arterial stiffness, impaired baroreflex sensitivity, aberrant neurohumoral regulation, and endothelial dysfunction all contribute to the modulation of BPV. These pathological alterations do not merely arise as consequences of erratic blood pressure; they also act as pivotal mechanisms in the initiation and progression of TOD (10). Evidence from multiple studies (11, 12) suggests that elevated BPV can directly induce structural and functional impairment in vital organs including the heart, brain, and kidneys. It does so by raising shear stress on vascular walls, promoting oxidative stress and inflammatory cascades, and accelerating the development of atherosclerosis. Accordingly, BPV may represent more than just a marker of cardiovascular risk; it likely functions as an independent risk factor actively involved in the pathogenesis of target organ injury.

In recent years, a considerable number of epidemiological and clinical investigations have focused on the relationship between short-term BPV and TOD. Multiple studies have indicated (13, 14) that 24-h systolic blood pressure SD and CV are closely associated with left ventricular hypertrophy, carotid atherosclerosis, and declining renal function. Abnormal nocturnal blood pressure dipping has also been linked to a higher risk of target organ injury, implying that nighttime blood pressure fluctuation patterns carry unique significance in protecting target organs. Nevertheless, the predictive value of different BPV indices varies across different forms of TOD, and findings across studies are not entirely consistent (15). Among various short-term BPV parameters, SD remains the most widely used measure of variability, reflecting the degree to which blood pressure deviates from its mean level. CV, expressed as the ratio of SD to mean blood pressure, helps reduce the confounding influence of average blood pressure levels on variability (16). ARV, calculated as the average of absolute differences between successive readings, better captures beat-to-beat fluctuations in blood pressure. Despite these well-established metrics, consistent conclusions regarding the distinct predictive ability of individual BPV indices for TOD remain lacking in current literature.

Most existing research has been conducted in European and American populations, whereas studies focusing on the characteristics of BPV and its association with TOD among Chinese individuals remain relatively limited (17). Hypertensive patients in China may exhibit distinct blood pressure fluctuation patterns, including a higher prevalence of salt-sensitive hypertension and more frequent nocturnal non-dipping patterns. These ethnic and phenotypic differences may potentially alter the relationship between BPV and TOD (18). Against this background, systematically evaluating the independent predictive value of short-term BPV parameters for subclinical TOD in Chinese patients with essential hypertension, and clarifying the predictive performance of different indices across various organ systems, carries substantial clinical significance. Such evidence can help refine risk stratification and support the development of more individualized therapeutic strategies for hypertensive patients in the Chinese population.

This study adopted a retrospective cohort design. By analyzing 24-h ABPM data from 176 patients with essential hypertension, we systematically evaluated the predictive value of short-term BPV indices—including SD, CV, and ARV of 24-h, daytime, and nighttime systolic and diastolic blood pressure—for subclinical TOD. The primary aim was to identify the optimal predictive indices and their cut-off values, thereby providing a novel assessment tool and intervention target for the protection of target organs in hypertensive patients.

## 2 Patients and methods

### 2.1 Study design

**Figure 1:**
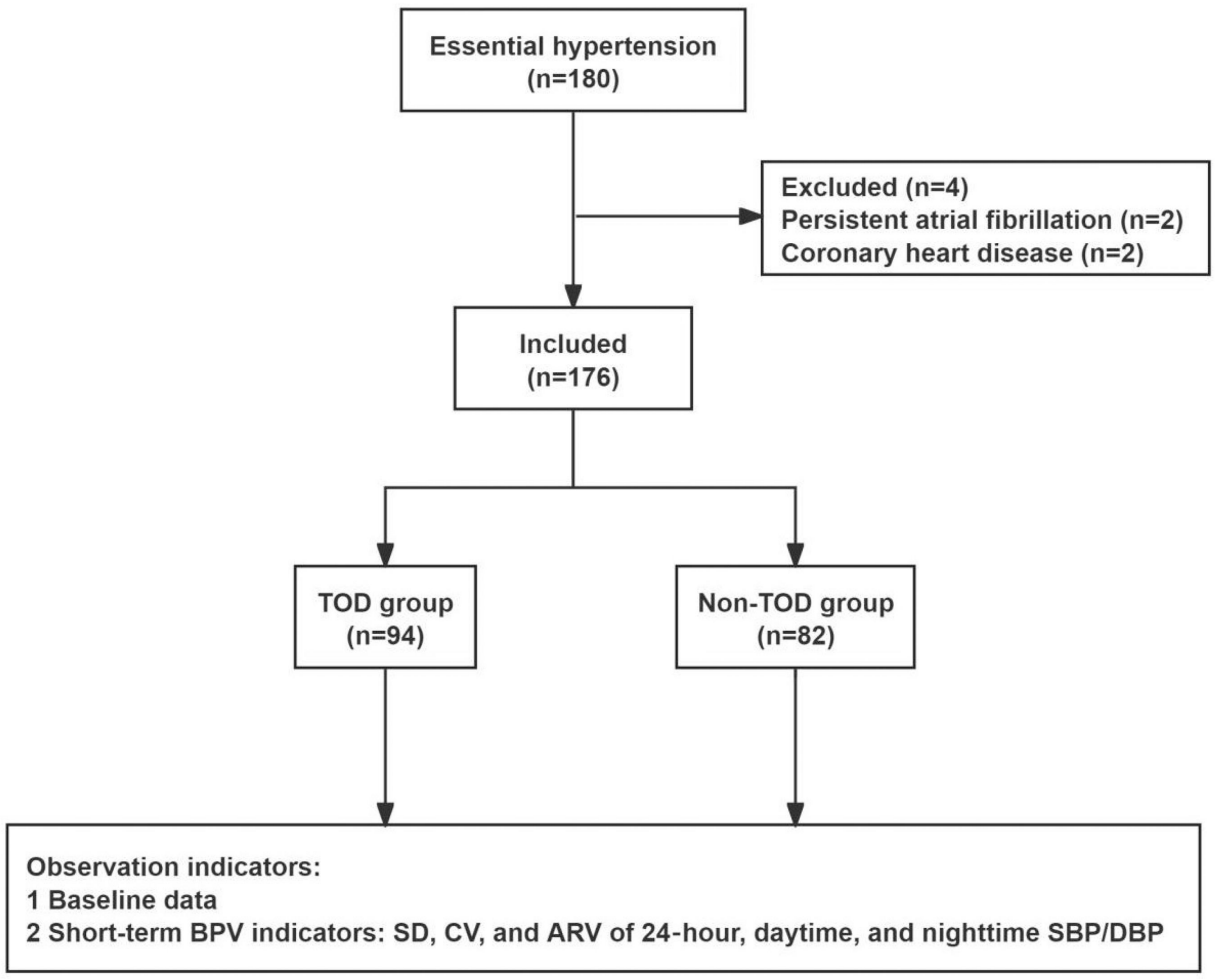
Flowchart of the study. A total of 180 patients with essential hypertension were initially screened. Of these, 4 patients were excluded due to persistent atrial fibrillation (*n* = 2) or coronary heart disease (*n* = 2). The remaining 176 eligible patients were divided into the TOD group (*n* = 94) and the non-TOD group (*n* = 82). The observation indicators included baseline clinical characteristics and short-term BPV indices, namely SD, CV, and ARV of 24-h, daytime, and nighttime SBP and DBP. TOD, target organ damage; BPV, blood pressure variability; SD, standard deviation; CV, coefficient of variation; ARV, average real variability; SBP, systolic blood pressure; DBP, diastolic blood pressure.

We carried out a retrospective cohort study in this research. A total of 180 patients diagnosed with essential hypertension and treated at Quanzhou First Hospital between January 2023 and December 2025 were initially screened. After applying exclusion criteria, 176 subjects were ultimately included in the final analysis. Based on the presence or absence of subclinical TOD, these participants were divided into two groups: the TOD-positive group and the TOD-negative group, as illustrated in Figure 1.

### 2.2 Inclusion criteria

(1) Patients who received medical care at Quanzhou First Hospital between January 2023 and December 2025, with a confirmed diagnosis of essential hypertension. (2) Met the diagnostic criteria for essential hypertension as defined in the Chinese Guidelines for the Prevention and Treatment of Hypertension (2024 Revision) (19). Specifically, this meant either: untreated individuals with three separate clinic measurements on different days showing a systolic blood pressure of 140 mmHg or higher and/or a diastolic blood pressure of 90 mmHg or above; or those already diagnosed with essential hypertension and on regular antihypertensive therapy. (3) Had complete 24-h ABPM data available in medical records, with acceptable monitoring quality defined as valid readings exceeding 80%, at least 20 daytime readings, no fewer than 7 nighttime readings, and total monitoring duration of at least 20 h. (4) Underwent a full set of examinations for subclinical TOD during the same period, including echocardiography (with assessment of left ventricular mass index), carotid ultrasound (with measurement of intima-media thickness), renal function tests [including serum creatinine (Scr), estimated glomerular filtration rate (eGFR) and urinary albumin-to-creatinine ratio (UACR)]. All renal abnormalities (reduced eGFR and elevated UACR) used to define renal subclinical TOD were required to persist for at least 3 months based on serial laboratory records. (5) Possessed complete clinical information retrievable from the electronic medical record system, covering demographic characteristics, past medical history, medication history, physical examination findings, and laboratory test results. (6) Aged between 18 and 75 years old. (7) Completed additional standardized examinations for comprehensive subclinical TOD screening, including transthoracic echocardiography for thoracic aortic diameter measurement, abdominal ultrasound for abdominal aortic evaluation, and fundoscopy for hypertensive retinopathy assessment.

### 2.3 Exclusion criteria

(1) Patients with a clear diagnosis of secondary hypertension documented in medical records. (2) Individuals accompanied by severe cardiac arrhythmias, such as persistent atrial fibrillation, frequent ventricular premature beats, or atrioventricular block of second degree or higher. (3) Those with established clinical cardiovascular diseases including coronary heart disease, heart failure, stroke, and peripheral arterial disease, as well as patients with end-stage renal disease defined by an estimated glomerular filtration rate (eGFR) < 15 mL·min^−1^·1.73m^−2^ (20). (4) Subjects who had their antihypertensive medication regimens modified within three months prior to ABPM examination. (5) Patients under acute stress conditions including acute infection, trauma, or surgery, or those complicated with severe hepatic or renal dysfunction, malignant tumors, or active autoimmune diseases. (6) Women who were pregnant or breastfeeding. (7) Cases with missing key data that made it impossible to calculate BPV parameters or evaluate the status of TOD. (8) Patients with transient, non-persistent eGFR decline or transient elevated UACR without a documented abnormal duration of ≥3 months, who could not be classified as having renal subclinical TOD.

### 2.4 Data collection

Clinical information of patients with essential hypertension treated at Quanzhou First Hospital between January 2023 and December 2025 was retrospectively collected from the hospital's electronic medical record system and laboratory information system. Data extracted covered demographic characteristics, medical histories, raw 24-h ABPM recordings, and results of laboratory examinations. The time interval between ABPM assessment and TOD evaluation was restricted to no more than two weeks. In addition, patients were required to maintain stable antihypertensive regimens for at least three consecutive months before testing, with no changes in drug categories or dosages. All relevant examinations were performed under conditions of stable blood pressure control. Two trained investigators independently extracted data using a standardized case report form, and all records were double-entered into the research database to ensure accuracy and consistency.

### 2.5 Antihypertensive treatment regimen

All enrolled patients received standardized oral antihypertensive pharmacotherapy. Treatment plans were formulated individually by attending cardiologists, in accordance with recommendations outlined in the Chinese Guidelines for the Prevention and Treatment of Hypertension (2024 Revision) (19). Medication types and corresponding dosages remained unchanged throughout the study period, which spanned from three months prior to enrollment until the completion of TOD assessment.

Commonly used antihypertensive agents included the following preparations. Calcium channel blockers (CCB) consisted of amlodipine besylate tablets (approval number: H20020390, strength: 5 mg per tablet, manufactured by Pfizer Pharmaceuticals Co., Ltd., Dalian, Liaoning Province), administered at a daily dose of 5–10 mg once daily (21). Angiotensin-converting enzyme inhibitors (ACEI) included perindopril erbumine tablets [approval number: H20034053, strength: 4 mg per tablet, produced by Servier (Tianjin) Pharmaceuticals Co., Ltd., Tianjin], with a usual daily dosage of 4–8 mg once daily. Angiotensin II receptor blockers (ARB) were represented by valsartan capsules (approval number: H20040217, strength: 80 mg per capsule, manufactured by Novartis Pharmaceuticals Co., Ltd., Beijing), given at 80–160 mg once daily. Beta-blockers included metoprolol succinate extended-release tablets (approval number: J20150044, strength: 47.5 mg per tablet, produced by AstraZeneca Pharmaceuticals Co., Ltd., Wuxi, Jiangsu Province), prescribed at 47.5–95 mg once daily (22). The thiazide-like diuretic used was indapamide tablets (approval number: H20083827, strength: 2.5 mg per tablet, manufactured by Tianjin Lisheng Pharmaceutical Co., Ltd., Tianjin), administered at 1.25–2.5 mg once daily. For patients on combination therapy, detailed doses and specific drug combinations were documented individually. Participants who required any addition, discontinuation, or dosage adjustment of antihypertensive medications during the study period were excluded from further analysis. This strict exclusion criterion was applied to ensure that assessments of BPV would not be confounded by changes in pharmacotherapy.

### 2.6 Outcome measures

#### 2.6.1 Twenty-four-hour ABPM

Twenty-four-hour ABPM was performed using a portable ABPM device (CB-1805-B; Wuxi Zhongjian Keyi Co., Ltd.; Jiangsu Province, China). The device was calibrated before each test, with the cuff placed on the dominant upper arm of each participant.Blood pressure readings were automatically obtained at 20-min intervals during daytime hours (06:00–22:00) and at 30-min intervals during nighttime hours (22:00–06:00), with systolic blood pressure, diastolic blood pressure, and heart rate all recorded simultaneously. A valid ABPM dataset was defined as a total monitoring duration of at least 20 h, with more than 80% of readings deemed valid (≥20 valid readings during the day and ≥7 at night). Artifacts and technically disturbed measurements were excluded from further processing. Indices of BPV were calculated using the manufacturer's dedicated software [ABP VERSION 9.1.0(2018-10-21)]. SD was used to reflect the dispersion of BP readings across the recording period. CV was expressed as the ratio of SD to the corresponding mean blood pressure. ARV was computed as the mean of absolute differences between successive BP measurements (23).

#### 2.6.2 Cardiac structure and function assessment

Echocardiographic examinations were carried out using a color Doppler ultrasound system (Vivid E95, GE Healthcare, Milwaukee, WI, USA) equipped with an M5Sc-D transducer operating at a frequency range of 1.5–4.6 MHz. All participants were examined in the left lateral decubitus position with continuous electrocardiographic gating. Interventricular septal thickness (IVST), left ventricular posterior wall thickness (LVPWT), and left ventricular internal diameter at end-diastole (LVIDd) were measured from the parasternal long-axis view. Left ventricular ejection fraction (LVEF) was determined via the biplane Simpson method. The early (*E*) and late (*A*) diastolic mitral inflow velocities were recorded by pulsed-wave Doppler, while tissue Doppler imaging was used to measure early diastolic mitral annular velocity (*e*′), allowing calculation of the *E*/*e*′ ratio. Left ventricular mass index (LVMI) was calculated according to the Devereux formula: LVM = 0.8 × 1.04 × [(IVST + LVIDd + LVPWT)^3^ − LVIDd^3^] + 0.6; LVMI = LVM/body surface area. Left ventricular hypertrophy was defined as an LVMI ≥115 g/m^2^ in men and ≥95 g/m^2^ in women (24).

#### 2.6.3 Vascular structure and function assessment

Carotid intima-media thickness (CIMT) was measured with a high-resolution ultrasound system (LOGIQ E20, GE Healthcare, Milwaukee, WI, USA) using an ML6-15 linear array transducer at a frequency range of 6–15 MHz. Each subject was examined in the supine position with the head turned slightly away from the side being scanned. Measurements were obtained on the posterior wall 1.0 cm proximal to the carotid bulb, and the final value was taken as the average of three consecutive cardiac cycles. Vascular damage was defined as a CIMT ≥ 0.9 mm or the presence of any atherosclerotic plaque. Brachial-ankle pulse wave velocity (baPWV) was determined using an automated arteriosclerosis analyzer (BP-203RPE III, Omron Healthcare Co., Ltd., Kyoto, Japan). After resting quietly in the supine position for 10 min, oscillometric blood pressure cuffs were applied to all four limbs, and baPWV was recorded automatically.Arterial stiffness was defined as a baPWV value >1,400 cm/s (25).

#### 2.6.4 Renal function assessment

Blood samples were collected and analyzed for Scr using an enzymatic assay on a fully automated clinical chemistry analyzer (cobas c 702, Roche Diagnostics, Mannheim, Germany). eGFR was calculated using the CKD-EPI equation. First-morning voided urine samples were collected for measurement of urinary microalbumin (UMA) via an immunoturbidimetric method, while urinary creatinine (UCr) was determined by an enzymatic creatinine assay. The UACR was then computed. All urinary measurements were performed using commercial kits (Lot: B0H181, Roche Diagnostics, Mannheim, Germany). Renal damage was defined as a UACR ≥ 30 mg/g (26).

### 2.7 Ethical statement

This study was performed in strict accordance with the principles outlined in the Declaration of Helsinki. All research procedures were reviewed and approved by the Ethics Committee of Quanzhou First Hospital, and informed consent was waived (Ethics Approval No.: [2026]K176).

### 2.8 Sample size calculation

As a retrospective cohort study, the sample size was determined with reference to previous investigations (27). In patients with TOD, the SD of nighttime systolic blood pressure was reported as 14.5 ± 4.0 mmHg, while in those without TOD it was 12.0 ± 3.5 mmHg. Using G*Power software, the corresponding Cohen's d effect size was calculated to be approximately 0.67. With a two-sided type I error rate *α* = 0.05 and statistical power (1 − *β*) set at 0.80, the minimum required sample size was 76 subjects per group, yielding a total of 152 patients. Accounting for an anticipated 5%–10% rate of missing data, we aimed to recruit at least 80 participants per group, for an overall target of 160 cases. In the final analysis, 176 patients were included (94 in the TOD group and 82 in the non-TOD group), which exceeded the required sample size. Post-hoc power analysis was performed for the SD of nighttime systolic blood pressure. The observed Cohen's d effect size in the present dataset was 5.318. At the current sample size and with *α* = 0.05 (two-sided), the achieved statistical power was 0.99 (99%), considerably higher than the pre-specified value of 0.80.This result confirms that the study possesses sufficient statistical power for evaluating the primary endpoint.

### 2.9 Statistical analysis

A logistic regression model was constructed to adjust for potential confounding factors, including age, sex, body mass index (BMI), duration of hypertension, 24-h mean systolic blood pressure, diabetes mellitus status, smoking history, number of antihypertensive drug classes, and statin use. Normality was assessed using the Shapiro-Wilk test, and homogeneity of variances was evaluated with Levene's test prior to group comparisons. Normally distributed continuous data were presented as mean ± SD and compared between groups using independent samples *t*-tests. Non-normally distributed variables were expressed as median (interquartile range) [M (P25, P75)] and analyzed with the Mann–Whitney *U* test. Categorical data were summarized as frequencies (percentages) [*n* (%)] and compared using the chi-square test or Fisher's exact test where appropriate. Multivariable logistic regression analysis was applied to evaluate the independent predictive value of various BPV indices for subclinical TOD, with results reported as odds ratios (OR) and 95% confidence intervals (CI). Receiver operating characteristic (ROC) curve analysis was performed to assess the predictive performance of each BPV parameter, including calculation of the area under the curve (AUC), optimal cutoff value, sensitivity, and specificity. Differences in AUCs between indices were compared using the DeLong test. A two-sided *P* < 0.05 was considered statistically significant.

## 3 Results

### 3.1 Baseline data

Table 1 presents a comparison of baseline clinical characteristics between patients with TOD (TOD group, *n* = 94) and those without evidence of TOD (non-TOD group, *n* = 82). The mean age in the TOD group was 60.89 ± 6.50 years, while the non-TOD group had an average age of 61.05 ± 6.20 years; no statistically significant difference was observed between the two groups (*P* = 0.871, OR = 1.004, 95% CI: 0.958–1.052). Gender distribution was well balanced across groups, with males accounting for 51.1% and 51.2% respectively (*P* = 0.984, OR = 1.006, 95% CI: 0.556–1.820). No significant between-group differences were detected in terms of BMI (26.31 ± 1.02 vs. 26.18 ± 0.86 kg/m^2^; *P* = 0.373, OR = 0.866, 95% CI: 0.632–1.188), duration of hypertension (8.27 ± 2.01 vs. 8.09 ± 2.02 years; *P* = 0.551, OR = 0.956, 95% CI: 0.824–1.109), or 24-h mean systolic blood pressure (135.63 ± 1.23 vs. 135.59 ± 1.14 mmHg; *P* = 0.813, OR = 0.970, 95% CI: 0.755–1.247). Similarly, the prevalence of diabetes mellitus (29.8% vs. 28.0%; *P* = 0.800, OR = 0.919, 95% CI: 0.478–1.767), current smoking status (23.4% vs. 17.1%; *P* = 0.301, OR = 0.674, 95% CI: 0.319–1.423), number of antihypertensive drug classes used (1.95 ± 0.72 vs. 1.82 ± 0.82; *P* = 0.264, OR = 0.801, 95% CI: 0.543–1.182), and the proportion of patients receiving statin therapy (36.2% vs. 30.5%; *P* = 0.426, OR = 0.774, 95% CI: 0.412–1.455) showed no meaningful statistical differences between the two cohorts. Overall, baseline profiles of patients in both groups were highly comparable. All *P*-values were greater than 0.05, and the 95% CI for all ORs included 1.0. These findings indicate that the baseline variables assessed did not exert a significant confounding effect on the observed differences in the occurrence of TOD.

**Table 1:** Baseline clinical data.

Indicators	TOD group (*n* = 94)	Non-TOD group (*n* = 82)	*P*	OR	95% CI for OR
Age (years, mean ± SD)	60.89 ± 6.50	61.05 ± 6.20	0.871	1.004	0.958, 1.052
Gender (*n* %)			0.984	1.006	0.556, 1.820
Male	48 (51.1)	42 (51.2)			
Female	46 (48.9)	40 (48.8)			
BMI (kg/m^2^, mean ± SD)	26.31 ± 1.02	26.18 ± 0.86	0.373	0.866	0.632, 1.188
Duration of hypertension (years, mean ± SD)	8.27 ± 2.01	8.09 ± 2.02	0.551	0.956	0.824, 1.109
24-h mean SBP (mmHg, mean ± SD)	135.63 ± 1.23	135.59 ± 1.14	0.813	0.970	0.755, 1.247
Diabetes mellitus (*n* %)	28 (29.8)	23 (28.0)	0.800	0.919	0.478, 1.767
Smoking (*n* %)	22 (23.4)	14 (17.1)	0.301	0.674	0.319, 1.423
Antihypertensive drug classes (numbers, mean ± SD)	1.95 ± 0.72	1.82 ± 0.82	0.264	0.801	0.543, 1.182
Statins (*n* %)	34 (36.2)	25 (30.5)	0.426	0.774	0.412, 1.455

TOD, target organ damage; OR, odds ratio; 95% CI, 95% confidence interval; SD, standard deviation; SBP, systolic blood pressure; BMI, body mass index.

### 3.2 SD, CV, and ARV of 24-h SBP and DBP

Table 2 summarizes comparisons of 24-h BPV indices between patients with TOD and those without TOD. For 24-h systolic BPV, the SD in the TOD group was 14.90 ± 1.06 mmHg, which was markedly higher than 10.38 ± 1.13 mmHg in the non-TOD group (*P* < 0.001). The corresponding Cohen's *d* effect size was 4.133, with a 95% CI for the mean difference of 4.20–4.85 mmHg. The CV in the TOD group reached 10.62 ± 0.85%, considerably greater than 7.40 ± 0.91% in the control group (*P* < 0.001), yielding an effect size of 3.660 and a 95% CI of 2.95%–3.48%. Similarly, the ARV value was 10.06 ± 0.92 mmHg in the TOD group, as opposed to 6.99 ± 1.02 mmHg in the non-TOD group (*P* < 0.001), with an effect size of 3.161 and a 95% CI for the difference of 2.78–3.36 mmHg. When examining diastolic BPV over 24 h, the SD in the TOD group was 9.43 ± 0.66 mmHg, far exceeding 6.49 ± 0.55 mmHg in the non-TOD arm (*P* < 0.001). This comparison produced an effect size of 4.781, the largest among all measured indices, accompanied by a 95% CI of 2.76–3.12 mmHg. The CV was 7.80 ± 0.56% in the TOD group, significantly elevated relative to 5.10 ± 0.46% in the comparison group (*P* < 0.001), with an effect size of 5.250 and a 95% CI of 2.55%–2.86%. The ARV was 6.90 ± 0.56 mmHg in patients with TOD, in contrast to 4.75 ± 0.51 mmHg in those without TOD (*P* < 0.001), corresponding to an effect size of 4.012 and a 95% CI of 2.00–2.32 mmHg. Statistically significant between-group differences were detected across all 24-h BPV parameters (*P* < 0.001). Moreover, all effect sizes exceeded 3.0, implying that these divergences are clinically meaningful rather than merely statistically driven. Patients in the TOD group overall exhibited substantially larger fluctuations in 24-h blood pressure compared with individuals free of TOD.

**Table 2:** Comparison of 24 h SBP/DBP SD, CV and ARV (mean ± SD).

Indicators		TOD group (*n* = 94)	Non-TOD group (*n* = 82)	*P*	Effect size (Cohen's *d*)	95% CI of the difference
24 h SBP	SD (mmHg)	14.90 ± 1.06	10.38 ± 1.13	<0.001	4.133	4.20, 4.85
	CV (%)	10.62 ± 0.85	7.40 ± 0.91	<0.001	3.660	2.95, 3.48
	ARV (mmHg)	10.06 ± 0.92	6.99 ± 1.02	<0.001	3.161	2.78, 3.36
24 h DBP	SD (mmHg)	9.43 ± 0.66	6.49 ± 0.55	<0.001	4.781	2.76, 3.12
	CV (%)	7.80 ± 0.56	5.10 ± 0.46	<0.001	5.250	2.55, 2.86
	ARV (mmHg)	6.90 ± 0.56	4.75 ± 0.51	<0.001	4.012	2.00, 2.32

DBP, diastolic blood pressure; CV, coefficient of variation; ARV, average real variability.

### 3.3 Daytime SD, CV, and ARV of systolic and diastolic blood pressure

Table 3 summarizes the comparative findings for daytime BPV indices between patients with and without TOD. For daytime systolic BPV, patients in the TOD group exhibited a mean SD of 14.09 ± 1.08 mmHg, which was markedly higher than the 9.75 ± 0.83 mmHg observed in the non-TOD group (*P* < 0.001). The corresponding Cohen's d effect size was 4.459, with a 95% CI for the mean difference ranging from 4.05 to 4.63 mmHg. Similarly, the CV in the TOD group reached 10.00 ± 0.90%, considerably greater than the 7.02 ± 0.65% in the control arm (*P* < 0.001), yielding an effect size of 3.746 and a 95% CI of 2.75%–3.21%. The ARV value was also elevated in the TOD group, at 9.36 ± 0.94 mmHg vs. 6.60 ± 0.71 mmHg in those without TOD (*P* < 0.001), with an effect size of 3.292 and a 95% CI of 2.52–3.01 mmHg. Turning to diastolic BPV during daytime hours, the TOD group displayed an SD of 9.30 ± 0.67 mmHg, which was significantly higher than the 6.31 ± 0.59 mmHg in the non-TOD subgroup (*P* < 0.001). The effect size here was 4.731, with a 95% CI of 2.81–3.19 mmHg. The CV was 7.64 ± 0.59% in TOD patients, in contrast to 4.95 ± 0.51% in individuals free of TOD (*P* < 0.001); this represented the largest effect size (4.818) among all daytime indices, accompanied by a 95% CI of 2.52%–2.85%. The ARV was 6.46 ± 0.91 mmHg in the TOD group, again significantly higher than 4.61 ± 0.55 mmHg in the comparison group (*P* < 0.001), with an effect size of 2.437 and a 95% CI of 1.64–2.08 mmHg. Notably, all daytime BPV parameters differed significantly between the two groups (*P* < 0.001). Furthermore, all effect sizes were greater than 2.4, suggesting that these between-group differences carry notable clinical relevance. Collectively, these findings demonstrate that patients with TOD show substantially larger daytime blood pressure fluctuations relative to those without TOD.

**Table 3:** Comparison of daytime SBP/DBP SD, CV and ARV (mean ± SD).

Indicators		TOD group (*n* = 94)	Non-TOD group (*n* = 82)	*P*	Effect size (Cohen's *d*)	95% CI of the difference
Daytime SBP	SD (mmHg)	14.09 ± 1.08	9.75 ± 0.83	<0.001	4.459	4.05, 4.63
	CV (%)	10.00 ± 0.90	7.02 ± 0.65	<0.001	3.746	2.75, 3.21
	ARV (mmHg)	9.36 ± 0.94	6.60 ± 0.71	<0.001	3.292	2.52, 3.01
Daytime DBP	SD (mmHg)	9.30 ± 0.67	6.31 ± 0.59	<0.001	4.731	2.81, 3.19
	CV (%)	7.64 ± 0.59	4.95 ± 0.51	<0.001	4.818	2.52, 2.85
	ARV (mmHg)	6.46 ± 0.91	4.61 ± 0.55	<0.001	2.437	1.64, 2.08

### 3.4 Nighttime SD, CV, and ARV of systolic and diastolic blood pressure

Table 4 presents comparisons of nighttime BPV indices between the TOD and non-TOD groups. With respect to nighttime systolic BPV, the TOD group had an SD of 14.94 ± 1.10 mmHg, which was notably higher than the 10.00 ± 0.69 mmHg recorded in the non-TOD group (*P* < 0.001). The Cohen's d effect size reached 5.318—the largest value across all time intervals—with a 95% CI for the mean difference of 4.68–5.21 mmHg. The CV in the TOD group was 11.36 ± 0.85%, markedly elevated compared with 7.27 ± 0.55% in the non-TOD group (*P* < 0.001). This corresponded to an effect size of 5.639, the highest among all 18 indices, and a 95% CI of 3.89%–4.31%. Similarly, the ARV was 10.67 ± 0.84 mmHg in the TOD group, significantly greater than 6.91 ± 0.60 mmHg in patients without TOD (*P* < 0.001), with an effect size of 5.100 and a 95% CI of 3.54–3.97 mmHg. For nighttime diastolic BPV, the TOD group showed an SD of 10.77 ± 1.08 mmHg, which was considerably higher than the 6.95 ± 0.80 mmHg seen in the non-TOD group (*P* < 0.001). The effect size was 3.973, with a 95% CI of 3.54–4.10 mmHg. The CV was 8.96 ± 0.98% in the TOD group, as opposed to 5.47 ± 0.74% in the non-TOD subgroup (*P* < 0.001), yielding an effect size of 3.970 and a 95% CI of 3.23%–3.74%. The ARV value reached 7.88 ± 0.96 mmHg in the TOD group, again significantly above the 4.99 ± 0.76 mmHg observed in the comparison group (*P* < 0.001), with an effect size of 3.313 and a 95% CI of 2.63–3.15 mmHg. All nighttime BPV indices demonstrated statistically significant differences between the two groups (*P* < 0.001), with all effect sizes exceeding 3.3. In particular, the effect size for nighttime systolic CV was as high as 5.639. These results imply that nighttime blood pressure fluctuations—especially systolic pressure variation—serve as the most sensitive indicator for distinguishing patients with TOD from those without. Overall, patients in the TOD group exhibited significantly greater nighttime blood pressure fluctuations compared with the non-TOD group.

**Table 4:** Comparison of nighttime SBP/DBP SD, CV and ARV (mean ± SD).

Indicators		TOD group (*n* = 94)	Non-TOD group (*n* = 82)	*P*	Effect size (Cohen's *d*)	95% CI of the difference
Nighttime SBP	SD (mmHg)	14.94 ± 1.10	10.00 ± 0.69	<0.001	5.318	4.68, 5.21
	CV (%)	11.36 ± 0.85	7.27 ± 0.55	<0.001	5.639	3.89, 4.31
	ARV (mmHg)	10.67 ± 0.84	6.91 ± 0.60	<0.001	5.100	3.54, 3.97
Nighttime DBP	SD (mmHg)	10.77 ± 1.08	6.95 ± 0.80	<0.001	3.973	3.54, 4.10
	CV (%)	8.96 ± 0.98	5.47 ± 0.74	<0.001	3.970	3.23, 3.74
	ARV (mmHg)	7.88 ± 0.96	4.99 ± 0.76	<0.001	3.313	2.63, 3.15

### 3.5 Multivariable logistic regression analysis for independent predictive value of Various BPV parameters in identifying TOD

Multivariable logistic regression analysis demonstrated that only selected BPV indices retained independent predictive significance for subclinical TOD, after adjustments were introduced for age, sex, BMI, duration of hypertension, 24-h mean systolic blood pressure, diabetes mellitus status, smoking history, number of antihypertensive drug classes, and statin administration. Within 24-h systolic blood pressure parameters, ARV emerged as an independent predictor (*β* = 0.270, *P* = 0.006; OR = 1.31, 95% CI: 1.08–1.59). In contrast, neither SD (*β* = 0.068, *P* = 0.108; OR = 1.07, 95% CI: 0.99–1.16) nor CV (*β* = 0.052, *P* = 0.368; OR = 1.05, 95% CI: 0.94–1.18) showed independent predictive capacity. All 24-h diastolic blood pressure indices, including SD, CV, and ARV, failed to reach statistical significance (all *P* > 0.05). Among daytime systolic and diastolic blood pressure measures, only daytime systolic ARV exhibited borderline significance (*β* = 0.185, *P* = 0.068; OR = 1.20, 95% CI: 0.99–1.46), yet this association did not meet conventional thresholds for statistical significance. No other daytime BPV parameters demonstrated independent predictive value. For nighttime systolic blood pressure, both SD (*β* = 0.215, *P* = 0.001; OR = 1.24, 95% CI: 1.09–1.41) and ARV (*β* = 0.247, *P* = 0.014; OR = 1.28, 95% CI: 1.05–1.56) qualified as independent predictors of subclinical TOD, whereas CV remained non-significant (*β* = 0.078, *P* = 0.208; OR = 1.08, 95% CI: 0.96–1.22). None of the nighttime diastolic blood pressure indices were identified as independent predictors (Table 5). Collectively, nighttime systolic SD, 24-h systolic ARV, and nighttime systolic ARV serve as independent predictors of subclinical TOD. Among these markers, nighttime systolic BPV indices display the most prominent predictive performance, implying that nocturnal blood pressure fluctuations play a critical role in the pathogenesis of TOD.

**Table 5:** Multivariate logistic regression analysis for evaluating the independent predictive value of different BPV indicators for TOD.

Indicators		*β*	*P*	OR	95% CI for OR
24 h SBP	SD (mmHg)	0.068	0.108	1.07	0.99, 1.16
	CV (%)	0.052	0.368	1.05	0.94, 1.18
	ARV (mmHg)	0.270	0.006	1.31	1.08, 1.59
24 h DBP	SD (mmHg)	0.045	0.238	1.05	0.97, 1.13
	CV (%)	0.038	0.432	1.04	0.95, 1.14
	ARV (mmHg)	0.112	0.208	1.12	0.94, 1.33
Daytime SBP	SD (mmHg)	0.058	0.198	1.06	0.97, 1.16
	CV (%)	0.042	0.445	1.04	0.94, 1.16
	ARV (mmHg)	0.185	0.068	1.20	0.99, 1.46
Daytime DBP	SD (mmHg)	0.035	0.392	1.04	0.95, 1.13
	CV (%)	0.028	0.588	1.03	0.93, 1.14
	ARV (mmHg)	0.095	0.272	1.10	0.93, 1.30
Nighttime SBP	SD (mmHg)	0.215	0.001	1.24	1.09, 1.41
	CV (%)	0.078	0.208	1.08	0.96, 1.22
	ARV (mmHg)	0.247	0.014	1.28	1.05, 1.56
Nighttime DBP	SD (mmHg)	0.092	0.112	1.10	0.98, 1.23
	CV (%)	0.065	0.238	1.07	0.96, 1.19
	ARV (mmHg)	0.138	0.148	1.15	0.95, 1.39

### 3.6 ROC curve analysis for evaluating the predictive performance of BPV indices in TOD

**Figure 2:**
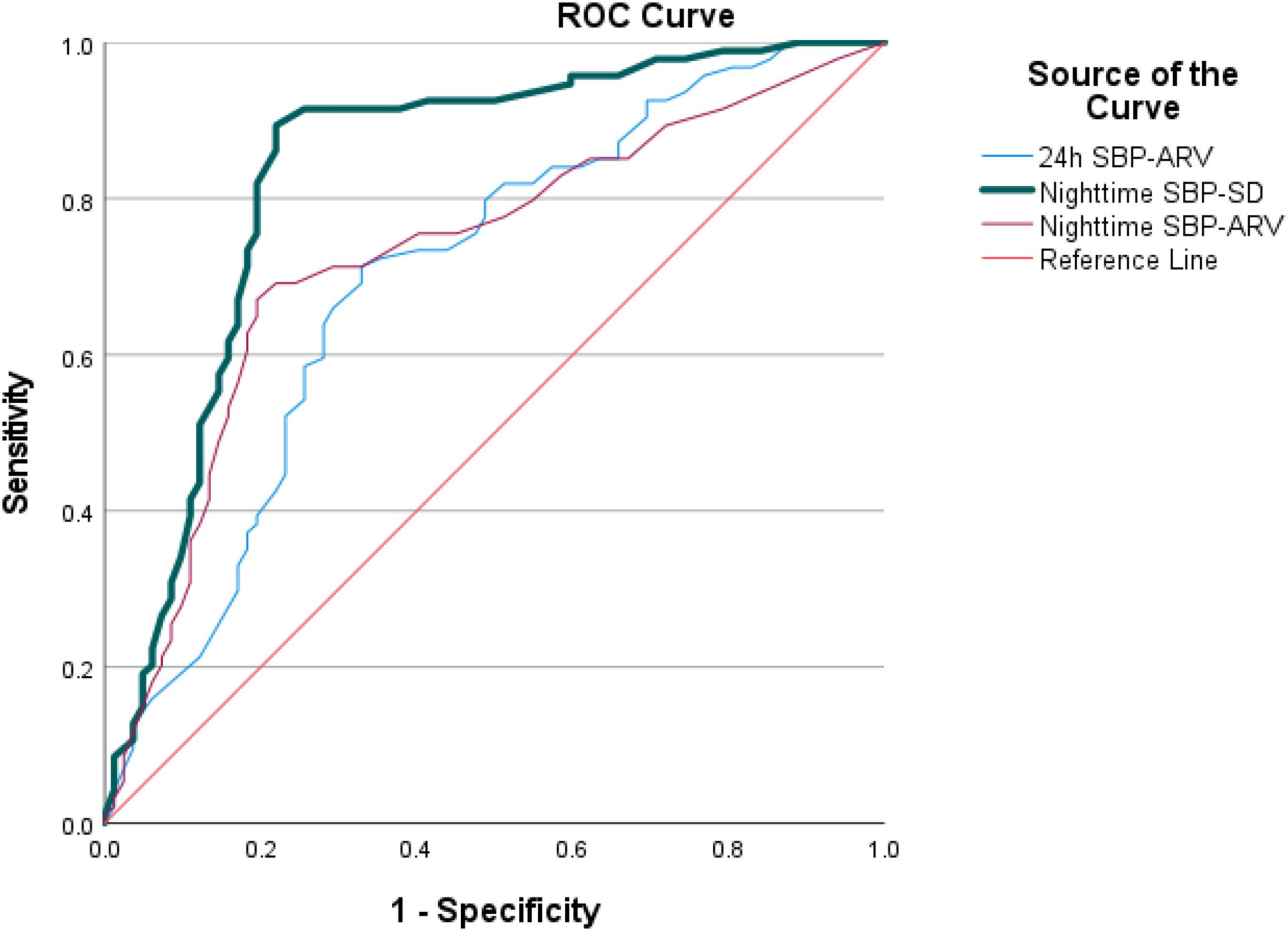
ROC curve for evaluating the predictive efficacy of BPV indicators for TOD. The ROC curve illustrates the predictive efficacy of 24-h and nighttime systolic blood pressure (SBP) variability indices for TOD. ROC, receiver operating characteristic.

Findings from Figure 2 and Table 6 demonstrate that the SD of nocturnal systolic blood pressure yields the strongest predictive capacity, with an AUC of 0.837 (95% CI: 0.773–0.901). The optimal cutoff value was determined to be 12.25 mmHg, corresponding to a sensitivity of 89.4% and a specificity of 78.0%. The Hosmer–Lemeshow test yielded a *P*-value of 0.254, supporting satisfactory goodness-of-fit for the constructed model. For the 24-h ARV of systolic blood pressure, the AUC was calculated as 0.701 (95% CI: 0.623–0.780), with an optimal cutoff of 8.65 mmHg, a sensitivity of 66.0%, and a specificity of 70.7%. The corresponding Hosmer–Lemeshow *P*-value stood at 0.373, indicating an acceptable level of model fitting. Meanwhile, nocturnal systolic ARV achieved an AUC of 0.730 (95% CI: 0.654–0.806), with a cutoff of 8.7 mmHg, a sensitivity of 67.0%, and a specificity of 80.5%; the Hosmer–Lemeshow *P*-value of 0.791 further verified adequate model fitness. Results from the DeLong test revealed that the AUC for nocturnal systolic BP SD was statistically higher than those for both 24-h systolic ARV (*P* = 0.003) and nocturnal systolic ARV (*P* = 0.007). This observation suggests that nocturnal systolic SD possesses a markedly superior discriminatory ability for subclinical TOD compared with the other two independent predictors. Given its high sensitivity of 89.4% and specificity of 78.0%, nocturnal systolic SD emerges as the most reliable single BPV parameter for identifying patients at high risk of subclinical TOD.

**Table 6:** Diagnostic efficacy indicators of ROC curve.

Indicators	Cut-off	Sensitivity (%)	Specificity (%)	AUC	95% CI for AUC	Delong (*P*)	Hosmer–Lemeshow (*P*)
24 h SBP-ARV	≥8.65 mmHg	66.0	70.7	0.701	0.623–0.780	0.003	0.373
Nighttime SBP-SD	≥12.25 mmHg	89.4	78.0	0.837	0.773–0.901	-	0.254
Nighttime SBP-ARV	≥8.7 mmHg	67.0	80.5	0.730	0.654–0.806	0.007	0.791

Delong (*P*) vs. nighttime SBP-SD; ROC, receiver operating characteristic; AUC, area under the curve.

### 3.7 Independent prognostic value of BPV parameters for different types of TOD

Multivariable logistic regression analysis was further applied to evaluate the independent predictive value of three candidate indicators—ARV of 24-h systolic BP, SD of nocturnal systolic BP, and ARV of nocturnal systolic BP—across distinct subtypes of TOD. In the subgroup with cardiac injury, all three indices emerged as independent predictors: ARV of 24-h systolic BP (*β* = 2.285, *P* = 0.004, OR = 9.830, 95% CI: 2.082–46.407), SD of nocturnal systolic BP (*β* = 2.799, *P* = 0.009, OR = 16.428, 95% CI: 2.017–133.805), and ARV of nocturnal systolic BP (*β* = 0.422, *P* = 0.042, OR = 1.525, 95% CI: 1.015–2.290). For vascular damage, ARV of 24-h systolic BP (*β* = 0.744, *P* < 0.001, OR = 2.104, 95% CI: 1.528–2.897) and SD of nocturnal systolic BP (*β* = 0.753, *P* = 0.003, OR = 2.124, 95% CI: 1.298–3.477) remained independently predictive, whereas ARV of nocturnal systolic BP failed to reach statistical significance (*P* = 0.894). In the renal impairment subgroup, only SD of nocturnal systolic BP demonstrated independent predictive utility (*β* = 0.568, *P* = 0.027, OR = 1.765, 95% CI: 1.068–2.918); neither ARV of 24-h systolic BP (*P* = 0.401) nor ARV of nocturnal systolic BP (*P* = 0.418) was significant (Table 7). Collectively, SD of nocturnal systolic BP conferred independent predictive capacity for cardiac, vascular, and renal TOD alike, marking it as the most broadly applicable marker. ARV of 24-h systolic BP showed strong predictive performance for cardiac injury but exhibited limited utility in identifying vascular or renal damage. By contrast, the predictive value of ARV of nocturnal systolic BP appeared inconsistent, suggesting potential organ-specific heterogeneity in its clinical relevance.

**Table 7:** Independent predictive value of BPV indices for different types of TOD.

Indicators		*β*	*P*	OR	95% CI for OR
Cardiac damage	Nighttime SBP-SD (mmHg)	2.799	0.009	16.428	2.017, 133.805
	24 h SBP-ARV (mmHg)	2.285	0.004	9.830	2.082, 46.407
	Nighttime SBP-ARV (mmHg)	0.422	0.042	1.525	1.015, 2.290
Vascular damage	Nighttime SBP-SD (mmHg)	0.753	0.003	2.124	1.298, 3.477
	24 h SBP-ARV (mmHg)	0.744	<0.001	2.104	1.528, 2.897
	Nighttime SBP-ARV (mmHg)	0.005	0.894	1.005	0.933, 1.083
Renal damage	Nighttime SBP-SD (mmHg)	0.568	0.027	1.765	1.068, 2.918
	24 h SBP-ARV (mmHg)	0.146	0.396	1.157	0.826, 1.619
	Nighttime SBP-ARV (mmHg)	0.023	0.536	1.023	0.952, 1.099

## 4 Discussion

This retrospective investigation enrolled a total of 176 patients diagnosed with essential hypertension, with all participants undergoing 24-h ABPM. A comprehensive analysis was performed to evaluate how well short-term BPV indices could predict the presence of subclinical TOD. According to the findings of this study, subjects in the TOD group exhibited markedly higher values of SD, CV, and ARV for both systolic and diastolic blood pressure across 24-h, daytime, and nighttime periods, compared with those in the non-TOD subgroup. Such observations imply that elevated fluctuations in blood pressure may be closely linked to the development of early-stage target organ injury. In the subsequent multivariable Logistic regression analysis, after adjusting for a range of potential confounding variables, nighttime systolic blood pressure SD, 24-h systolic blood pressure ARV, and nighttime systolic blood pressure ARV remained independent predictors for subclinical TOD. ROC curve analysis further demonstrated that nighttime systolic blood pressure SD achieved the most favorable predictive performance among all included indices. Subgroup analyses additionally revealed that nighttime SBP SD provided independent predictive value for damage involving cardiac, vascular, and renal target organs alike. Meanwhile, 24-h SBP ARV also exerted an independent predictive effect with respect to cardiac and vascular impairment, respectively.

In our current study, nighttime systolic BPV was found to carry strong independent predictive significance, a finding that may be closely linked to the unique physiological nature of nocturnal blood pressure regulation. During normal sleep, the human body shifts toward reduced sympathetic nerve activity and heightened vagal tone, a state under which blood pressure is expected to undergo a physiologic decline (28). In patients with established TOD, however, such inherent regulatory mechanisms may become impaired. This disruption often leads to an insufficient nocturnal blood pressure dip or even a reverse-dipper pattern, which in turn gives rise to elevated nighttime blood pressure fluctuations (29). Sustained nighttime hypertension or increased variability may inflict persistent injury to critical end organs including the heart, brain, and kidneys. Potential pathways include elevated vascular shear stress, enhanced oxidative stress and inflammatory responses, as well as accelerated progression of atherosclerosis (30). Furthermore, nighttime BPV is less susceptible to external confounding influences. As such, it may serve as a more reliable indicator of intrinsic autonomic regulatory function and underlying vascular pathological conditions. For these reasons, its predictive value for TOD may well surpass that of daytime BPV.

As an indicator that quantifies the average absolute difference between successive blood pressure readings, ARV effectively captures rapid beat-to-beat fluctuations in blood pressure. By contrast, SD merely characterizes the degree to which blood pressure values scatter around their mean. In the present investigation, 24-h systolic blood pressure ARV demonstrated independent predictive value for cardiac and vascular injury, implying that short-term, rapid fluctuations in blood pressure may better reflect the risk of targeted organ damage than overall dispersion does. This observation may be linked to the inherent physiological nature of ARV: it identifies rapid dynamic patterns within blood pressure time series. Such abrupt fluctuations are believed to exert more intense mechanical stress on the vascular wall, thereby accelerating the development of TOD (31). Nevertheless, ARV showed no independent predictive significance for renal impairment. This divergent finding suggests that distinct target organs may differ in their susceptibility to various blood pressure fluctuation profiles. The pathogenesis of renal damage may be more closely associated with sustained blood pressure burden rather than transient, short-term variations.

As outlined in the position statement released by the European Society of Hypertension, short-term BPV shows significant correlations with left ventricular hypertrophy, carotid atherosclerosis, and renal dysfunction, and these associations exist independently of mean blood pressure levels (32). Findings from the current study, which demonstrate the independent predictive value of nighttime systolic blood pressure SD, are consistent with the above evidence, lending further support to the importance of systolic BPV in the evaluation of hypertensive TOD. The SHIP AHOY investigation (33), conducted among adolescent participants, failed to identify an independent predictive role for ABPM-derived BPV indices in relation to left ventricular hypertrophy. Instead, only diastolic blood pressure CV during wakefulness and heart rate reduction emerged as significant determinants of left ventricular mass index. Such discrepancies may be partly attributed to age-related characteristics across study populations. Adolescents typically exhibit vascular compliance and autonomic regulatory functions that differ fundamentally from middle-aged and elderly hypertensive individuals, which may alter the relationship pattern between BPV and target organ injury. Furthermore, while the aforementioned study relied primarily on CV as the key marker of BPV, the present analysis did not detect an independent predictive capacity for this metric. This observation implies that the predictive performance of distinct BPV parameters may vary across different population groups. A cross-sectional, single-center study (34) conducted in patients with chronic kidney disease documented that systolic blood pressure weighted standard deviation (wSD) was significantly associated with left ventricular hypertrophy, abnormal CIMT, reduced eGFR, and albuminuria. Additionally, wSD was found to increase linearly with advancing CKD stages. In the current research, however, ARV did not demonstrate independent predictive utility in the subgroup with renal impairment, whereas nighttime systolic SD remained a significant predictor. This divergence may reflect the predictive specificity of different BPV indices for distinct subtypes of TOD. The SD parameter quantifies the overall dispersion of blood pressure readings, whereas ARV places greater emphasis on short-term fluctuation characteristics. It is plausible that the development of renal injury is more closely linked to global blood pressure variation rather than rapid, beat-to-beat fluctuations.

The innovation of this study can be summarized as follows. First, we systematically compared the predictive performance of three distinct BPV metrics, namely SD, CV, and ARV, in identifying subclinical TOD. Our results confirmed that nighttime systolic blood pressure SD and ARV served as independent predictive factors, whereas CV did not exhibit such an independent association. These findings may offer valuable evidence to assist clinicians in selecting appropriate BPV indicators in clinical practice. Second, the present work simultaneously evaluated damage across three key target organs—the heart, blood vessels, and kidneys. It was observed that nighttime systolic BP SD provided independent predictive value for all three organ systems, while ARV was mainly predictive of cardiac and vascular injury. This suggests that different BPV parameters may vary in their predictive efficiency for specific TOD. Third, we determined the optimal cutoff value of nighttime systolic BP SD to be 12.25 mmHg, establishing a clinically applicable tool for identifying high-risk individuals. Last, this study verified the predictive significance of nighttime BPV among Chinese patients with essential hypertension, which may help address current research gaps regarding BPV and TOD in the Chinese population.

## 5 Limitations of the study and future research directions

This work carries several notable limitations worth noting. To begin with, given its retrospective design, the current investigation cannot establish a definitive causal link between BPV and TOD. Even after adjusting for a wide array of confounding variables, unmeasured factors—including sleep apnea and salt sensitivity, among others—may still exert an unaccounted influence on the final outcomes. Second, the overall sample size is somewhat modest. This is especially evident in subgroup analyses, where the number of cases for each type of target organ injury is relatively small. Such constraints may lower statistical power, meaning that some potential associations did not reach statistically significant levels in our analyses. Third, this was a single-center study conducted among patients recruited from only one hospital. This design naturally raises the possibility of selection bias, and the generalizability of our findings remains to be confirmed by future multi-center investigations. Fourth, 24-h ABPM was performed only once during the study period, which prevented us from evaluating long-term trajectories in BPV. Evidence suggests that visit-to-visit BPV may carry independent prognostic implications that were not captured here (35). Fifth, diurnal blood pressure patterns—dipper, non-dipper, and reverse-dipper phenotypes—were not incorporated as potential effect modifiers. It is plausible that the predictive strength of nocturnal BPV could be modified by an individual's circadian blood pressure profile, which was not accounted for in the present models.

For future research directions, prospective cohort studies or randomized controlled trials are warranted to clarify the causal relationship between BPV and TOD. Further investigations may also explore whether antihypertensive regimens that effectively reduce BPV can translate into tangible clinical benefits for organ protection. It would be valuable to combine multi-modal assessment tools—such as cardiac magnetic resonance imaging and pulse wave analysis—to better elucidate the pathophysiological mechanisms through which elevated BPV contributes to target organ injury. In addition, developing cardiovascular risk prediction models that integrate BPV parameters may help validate the incremental value of such markers in risk stratification. Finally, replicating these findings across diverse populations with varying ethnic backgrounds, age groups, and comorbidity profiles will be essential to confirm the broader applicability of our results.

## 6 Conclusion

Our findings suggest that SD of nocturnal systolic blood pressure, as assessed by ABPM, may yield the strongest predictive performance for subclinical TOD. In routine clinical management of hypertensive individuals, beyond merely monitoring mean blood pressure values, placing greater emphasis on the evaluation of BPV—especially fluctuations in nocturnal systolic pressure—could assist clinicians in identifying high-risk patients for target organ injury with improved accuracy, thereby supporting the development of personalized antihypertensive treatment strategies. Nevertheless, given the retrospective nature and single-center design of the present investigation, the conclusions drawn herein require further validation through well-designed prospective studies.

## Data Availability

The original contributions presented in the study are included in the article/Supplementary Material, further inquiries can be directed to the corresponding author.
